# Epidemiological and Molecular Investigation of *Dirofilaria repens*, *Dirofilaria immitis*, and *Acanthocheilonema reconditum* in Dogs in the Republic of Srpska (Bosnia and Herzegovina)

**DOI:** 10.3390/ani16152361

**Published:** 2026-08-02

**Authors:** Darko Despotovic, Nemanja M. Jovanovic, Tamas Petrovic, Ivona Subic, Andrea Radalj, Oliver Stevanovic, Katarina Nenadovic, Tamara Ilic

**Affiliations:** 1 PI Veterinary Institute of the Republic of Srpska “Dr Vaso Butozan”, 78000 Banja Luka, Bosnia and Herzegovina; darkodespotovicbl@gmail.com (D.D.); ivona.subic@virs-vb.com (I.S.); oliver.stevanovic@virs-vb.com (O.S.); 2 Department of Parasitology and Parasitic Diseases, Faculty of Veterinary Medicine, University of Belgrade, Bul. Oslobodjenja 18, 11000 Belgrade, Serbia; tamara@vet.bg.ac.rs; 3 Scientific Veterinary Institute “Novi Sad”, Rumenacki put 20, 21113 Novi Sad, Serbia; tomy@niv.ns.ac.rs; 4 Department of Microbiology and Immunology, Faculty of Veterinary Medicine, University of Belgrade, Bul. Oslobodjenja 18, 11000 Belgrade, Serbia; andrea.zoric@vet.bg.ac.rs; 5 Department of Animal Hygiene, Faculty of Veterinary Medicine, University of Belgrade, Bul. Oslobodjenja 18, 11000 Belgrade, Serbia; katarinar@vet.bg.ac.rs

**Keywords:** dog, *Dirofilaria immitis*, *Dirofilaria repens*, *Acanthocheilonema reconditum*, prevalence, risk factors, molecular diagnostics, Bosnia and Herzegovina

## Abstract

Canine filariosis is an emerging vector-borne parasitic disease of veterinary and public health importance, caused primarily by *D. immitis* and *D. repens*. The present study investigated the prevalence, risk factors, diagnostic performance of different methods, and molecular characteristics of filarial infections in dogs from the Republic of Srpska (Bosnia and Herzegovina). Blood samples from 418 dogs collected between 2020 and 2024 were examined using a modified Knott test, antigen enzyme-linked immunosorbent assay, and polymerase chain reaction-based methods. Overall, 21.3% of dogs were positive for at least one filarial species. *D. repens* was the most prevalent species (13.40%), followed by *D. immitis* (10.8%) and *A. reconditum* (1.4%). Coinfections with *D. immitis* and *D. repens* were detected in 3.35% of dogs, while triple infections occurred in 0.5%. Several individual and environmental factors were associated with an increased risk of infection, including poor body condition, mixed-breed status, inadequate parasite control, and specific movement histories. PCR showed the highest diagnostic performance for all detected species, outperforming both microscopy and antigen testing. Phylogenetic analysis revealed genetic diversity among the detected isolates and demonstrated their relationship to strains previously reported from other European countries. These findings confirm the endemic presence of canine filarial infections in the Republic of Srpska and emphasize the importance of molecular diagnostics, surveillance programs, and preventive measures for effective disease control.

## Simple Summary

Canine filarial infections are vector-borne parasitic diseases that affect dogs and may also pose a risk to humans. This study investigated the occurrence of three filarial parasites in 418 dogs from different regions of the Republic of Srpska using microscopic, serological, and molecular diagnostic methods. More than 20% of the dogs were infected, with *Dirofilaria repens* being the most common species, followed by *D. immitis* and *Acanthocheilonema reconditum*. Molecular methods showed the highest diagnostic accuracy. These findings provide valuable epidemiological data and support improved surveillance, screening, and prevention programs in dogs.

## 1. Introduction

Dirofilariosis is a vector-borne parasitic disease caused by filarioid nematodes of the genus *Dirofilaria* (Spirurida, Onchocercidae) and is transmitted by various mosquito species belonging to the family Culicidae (*Aedes*, *Anopheles*, *Culex*) [1,2,3]. Of all *Dirofilaria* species, *Dirofilaria immitis* and *D. repens* are recognized as the clinically most significant parasites of dogs, causing heartworm disease and subcutaneous dirofilariosis, respectively [4,5,6,7]. In addition to their veterinary importance, both parasites possess a zoonotic potential and represent a growing public health concern. Humans may serve as accidental hosts, in whom infection often presents atypically but may also lead to severe clinical outcomes [1,8,9].

Prior to this study, the epidemiological situation of canine dirofilariasis in the Republic of Srpska was unknown, while data from recent studies in Europe, as well as in the southeastern regions of Asia and Africa, showed that the prevalence of *D. immitis* and *D. repens* is increasing [7,10,11]. However, there are studies that report changes in the prevalence of both parasites, identifying newly emerging endemic areas, formerly endemic areas with increased prevalence or hyperendemic areas with declining prevalence [12,13,14]. The prevalence of these parasites in dogs may depend on seasonal factors, the biological characteristics of the parasite species and intermediate hosts, as well as geographical differences among regions within a single country [15,16,17]. An additional risk factor is the persistently high number of stray dogs in certain countries, which are not covered by preventive measures and therefore serve as reservoirs of the parasites [18,19,20,21].

The diagnosis of dirofilariosis relies on the application of various microscopic, serological, and molecular methods; each plays a role in the accurate detection and confirmation of parasite presence in dogs [22]. Microscopic diagnostic methods, such as the Knott test and the examination of blood smears stained with Giemsa or hematoxylin eosin, enable direct visualization of microfilariae in blood samples [23]. These methods may exhibit reduced sensitivity in detecting low microfilarial burdens, which can result in false-negative findings [24]. A particular challenge in microscopic identification is the morphological similarity to microfilariae of other apathogenic filarial species of the genus *Acanthocheilonema* (*A. reconditum* and *A. dracunculoides*) [25]. The diagnosis of dirofilariosis often relies on serological tests (immunofluorescence assay—IFA, immunochromatographic tests—ICT, and enzyme-linked immunosorbent assay—ELISA), which, despite their effectiveness in detecting antibodies and antigens against *D. immitis*, are associated with certain limitations. Mostly, these tests may be affected by cross-reactivity with related parasites (*D. repens*, *A. reconditum*, and *A. dracunculoides*), which can potentially lead to false-positive results and misdiagnosis [26].

Therefore, PCR is recognized as a powerful tool for the precise detection of filarial DNA in clinical samples. The effectiveness of molecular diagnostics depends on key principles that enable proper test design, optimization, and interpretation in laboratory settings [22,27], as well as on the influence of inhibitory factors present in the examined clinical materials. Primer selection represents a critical initial step, as it directly affects the specificity and efficiency of amplification. Previous studies have demonstrated the effectiveness of various primer sets targeting conserved regions of *Dirofilaria* DNA, such as the cytochrome oxidase subunit 1 (*COI*) gene [24].

In general, diagnosis of dirofilariosis in dogs requires a multidisciplinary approach in order to avoid false-negative and false-positive results [22]. In this context, particular importance is attributed to the implementation of standardized diagnostic procedures for the detection of canine filariosis, improved screening of imported dogs, and the promotion of preventive measures among veterinary practitioners and dog owners. Accordingly, the aim of the present study is, in dogs within the geographical area of the Republic of Srpska, to: (i) determine the presence and prevalence of filarial infections of public health importance; and (ii) perform molecular identification and haplotyping of the detected filarial species.

## 2. Materials and Methods

### 2.1. Ethics Aspects

The protocol of this study was approved by the Ethics Committee for the Protection and Welfare of Experimental Animals in Biomedical Research of the Faculty of Medicine, University of Banja Luka, Republic of Srpska, Bosnia and Herzegovina (approval No. 18/1.22-2/24-1, dated 6 February 2023). Informed consent was obtained from the owners prior to the initiation of the study.

### 2.2. Study Area and Animals

The study was conducted on 418 dogs older than one year from the epidemiological area of the Republic of Srpska (42°33′–45°16′ N and 16°11′–19°37′ E), which extends across the northwestern, northern, eastern, and southeastern parts of Bosnia and Herzegovina. The area is influenced by a temperate continental, mountainous, mountain–basin, and Mediterranean climate. Samples were collected during the period from April 2020 to December 2024 from regions whose centers are the largest cities of the Republic of Srpska: Banja Luka, Prijedor, Gradiška, Doboj, Bijeljina, Istočno Sarajevo, and Trebinje.

To investigate the prevalence of filarial infections in the Republic of Srpska, the study aimed to collect blood samples from at least 210 dogs, with a minimum of 30 dogs from each of the seven regions. This sample size was calculated to detect an expected prevalence of 5% at the level of the Republic of Srpska and 10% within each region, with a 95% confidence level. Dogs were selected randomly. Non-owned dogs were sampled in animal shelters, whereas owned dogs were sampled during visits to veterinary clinics.

In order to assess potential risk factors associated with filarial infections, data on individual animals and their environmental conditions were collected using a standardized questionnaire and analyzed accordingly.

Individual factors included sex, neutering status, coat length (short, medium, or long), age (≤2 years, >2–8 years, or >8 years), body weight (≤5 kg, >5–20 kg, >20–40 kg, or >40 kg), body condition (working, breeding, good, obese, or thin/underweight), breed status (purebred or mixed-breed), and the presence or absence of clinical signs at the time of examination.

Environmental and management-related factors included dog purpose (indoor pet, outdoor pet, hunting, or working dog), housing conditions (indoor alone, permanently outdoors, indoors with the owner, or shelter), and origin (born to the current owner, acquired at <3 months, acquired at 3–12 months, acquired as an adult > 1 year, or stray/abandoned). Travel history was classified as no travel, short trips within Bosnia and Herzegovina, prolonged stay within Bosnia and Herzegovina, travel abroad, or unknown. Other investigated factors included mosquito protection (chemical protection, nets, or none), feeding type (owner-prepared, commercial, or mixed), blood-sampling time (07:00–10:00, 10:00–16:00, or after 16:00), and vaccination status (rabies only, rabies plus DHPPL, or unvaccinated). Deworming and ectoparasite control were recorded as performed or not performed, while ectoparasite presence was assessed at the time of examination. Ownership status was classified as owned or unowned, and previous therapy was defined as treatment with macrocyclic lactones during the 12 months preceding sampling.

For the purpose of analyzing the presence and prevalence of filarial infections in the Republic of Srpska, blood samples were collected. From each dog, a total of 4 mL of blood was obtained (2 mL of EDTA-anticoagulated blood and 2 mL for serum samples) by venipuncture of the cephalic vein of the forelimb (*vena cephalica antebrachii*), in accordance with European legislation on animal welfare.

### 2.3. Modified Knott Test and Antigen Testing

The Knott test was performed according to the method described by Genchi et al. [28]. The stained sediment was examined at magnifications of 100× and 400× using a light microscope. Identification of microfilariae was performed by measuring their length and assessing the morphology of the head and tail [23].

Detection of antigens of adult female *Dirofilaria immitis* in the peripheral blood of dogs was carried out using an enzyme-linked immunosorbent assay (ELISA) with the Heartworm Antigen Test Kit PetChek^®^ HTWM PF (IDEXX, Hoofddorp, The Netherlands), which, according to the manufacturer, has a sensitivity of 98% and a specificity of 100%. The ELISA test was performed in accordance with the manufacturer’s instructions.

### 2.4. Molecular Diagnostics

For molecular identification of microfilarial species, genomic DNA was extracted from 200 microliters of each blood sample using the commercial IndiSpin Pathogen Kit (Qiagen, Hilden, Germany), in accordance with the manufacturer’s instructions. Detection of genomic DNA of *D. immitis*, *D. repens*, and *A. reconditum* was performed using the end-point PCR method, following the protocol described by Rishniw et al. [29]. Amplification of the target DNA sequences was carried out using DreamTaq™ Hot Start PCR Master Mix (2×) (Thermo Scientific, Fisher Scientific Oy, Vantaa, Finland).

In the first phase, panfilarial oligonucleotide primers were used for the detection of filarial DNA belonging to the family Onchocercidae (Table 1). Samples with positive findings were subsequently analyzed using species-specific primers for the identification of *D. immitis*, *D. repens*, and *A. reconditum* (Table 1). DNA isolated from morphologically and PCR-confirmed adult worms or microfilariae of the respective species was used as a positive control. PCR reactions were performed on an Eppendorf Mastercycler Nexus thermal cycler (Hamburg, Germany).

The obtained PCR products were separated by horizontal electrophoresis on a 1.5% agarose gel stained with Midori Green Direct dye (Nippon Genetics Europe, Düren, Germany), using a Sub-Cell GT Cell electrophoresis system with a PowerPac Basic Power supply (Bio-Rad, Hercules, CA, USA). Visualization of the amplified DNA fragments was carried out under UV light using a Hoefer Macro Vue UV-20 transilluminator (Pharmacia Biotech, Barcelona, Spain).

### 2.5. Sequencing and Phylogenetic Analysis

The segments of 5.8S-*ITS2* genes of *D. immitis*, *D. repens*, and *A. reconditum* (542, 578, and 484 bp) were subjected to PCR amplification [30]. The selected PCR products from the areas of Brod (*n* = 1), Gacko (*n* = 1), Kozarska Dubica (*n* = 1), Prijedor (*n* = 2), and Gradiska (*n* = 2) were sent for sequencing to Macrogen Europe (Amsterdam, The Netherlands). The samples were sequenced in both directions and analyzed using BLAST (version 2.15.0) [30] after building consensus sequences using MEGA7 software (version 7.0.26) [31]. The pairwise distances were calculated using MEGA7. Maximum Likelihood phylogenetic trees were constructed in MEGA7 using the Tamura-3-parameter model, supported by bootstrap analysis with 1000 reiterations. The obtained sequences were deposited to GenBank under the following accession numbers: PQ615124–PQ615126 for *D. immitis*, PQ615127 for *A. reconditum*, and PQ615128–PQ615130 for *D. repens*.

### 2.6. qGIS

The data were analyzed using the geographic information system QGIS version 3.36 [32] for visualization of the regional distribution of: (i) collected and analyzed blood and serum samples; (ii) samples positive for *D. immitis*; (iii) samples positive for *D. repens*; (iv) samples positive for both *D. immitis* and *D. repens*; and (v) samples positive for all three parasite species (*D. immitis*, *D. repens*, and *A. reconditum*). Sample locations were georeferenced using four-digit postal codes and displayed on maps with administrative boundaries. The maps also depict regional boundaries that are neither administrative nor official but are used solely to facilitate understanding of the geographical epidemiology in this study.

### 2.7. Statistical Analyses

Data on dogs collected through the questionnaire were entered into Microsoft Excel software version 2021 (Microsoft Corporation, Redmond, WA, USA). Statistical analysis was performed using IBM SPSS Statistics, version 28 (IBM Corp., Armonk, NY, USA). The level of statistical significance was set at *p* < 0.05. Results were presented descriptively as absolute frequencies (*n*) and relative proportions (%). The prevalence of infections was expressed with 95% confidence intervals (95% CI), calculated using the exact Clopper–Pearson method. Differences in prevalence between the examined categories were analyzed using Pearson’s χ^2^ test.

The effect of risk factors on the occurrence of infection was assessed by univariable logistic regression analysis. For each analyzed variable, the category with the lowest prevalence was defined as the reference category. In cases where zero cells occurred, the Haldane–Anscombe correction was applied when calculating odds ratios (OR) by adding 0.5 to all cells of the table. Variables that showed statistical significance or a trend toward significance (*p* ≤ 0.25) in the univariable analysis were included in the multivariable logistic regression model. Model results were presented as regression coefficients (β), standard errors (SE), odds ratios (OR), and their 95% confidence intervals.

Pairwise comparison of diagnostic methods for the detection of *D. immitis*—modified Knott test, HW Ag ELISA, species-specific PCR, and panfilarial PCR—was performed on the subset of dogs that tested positive by at least one of these methods. Differences in detection rates between paired methods were assessed using McNemar’s test, while the degree of agreement was analyzed using Cohen’s kappa coefficient (κ). The magnitude and direction of disagreement between methods were evaluated by calculating the matched odds ratio (mOR) and the corresponding 95% confidence interval based on the number of discordant pairs. Considering the possible influence of previous treatment with macrocyclic lactones on diagnostic results, all analyses—McNemar’s test, Cohen’s κ, and mOR—were repeated on the corrected dataset after excluding dogs that were positive exclusively by the HW Ag ELISA test.

## 3. Results

### 3.1. Prevalence and Regional Distribution of Canine Filariosis in the Republic of Srpska

**Figure 1:**
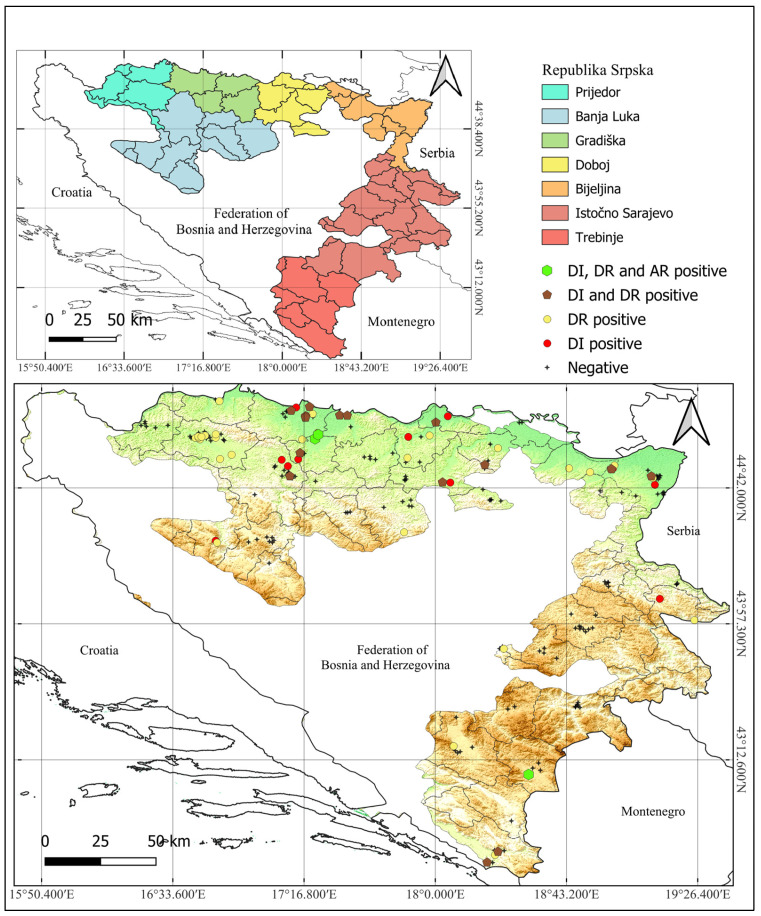
Locations of positive samples from tested dogs. Map was generated using qGIS [33].

Analysis of 418 canine blood samples revealed infections with three filarial species, with an overall prevalence of 21.3% (89/418). The most prevalent species was *D. repens* (13.4%), followed by *D. immitis* (10.8%), and *A. reconditum* (1.4%) (Table 2). Coinfection with *D. immitis* and *D. repens* was confirmed in 14 dogs (3.4%), while all three filarial species were detected in 2 dogs (0.5%). The highest prevalence of *D. immitis* (25.6%) and *A. reconditum* (6.1%) was recorded in the Gradiška region, whereas *D. repens* was most prevalent in the regions Prijedor (24%) and Gradiška (17.1%). The locations of positive samples from tested dogs are presented in Figure 1.

### 3.2. Risk Factor for D. immitis Infection

*Individual risk factors*. In the univariable analysis (Table 3), the risk of infection was significantly higher in thin dogs and dogs with breeding body condition compared with dogs in working body condition. Mixed-breed dogs had a higher risk of infection compared than purebred dogs. In addition, dogs showing clinical signs were more likely to be infected than asymptomatic dogs.

In the multivariable analysis, short-haired dogs showed an increased risk of infection than long-haired dogs. The higher risk was observed in underweight dogs and dogs with breeding body condition compared to dogs in working body condition. Also, mixed-breed dogs remained at significantly higher risk of infection than purebred dogs.

*Environmental risk factors*. In the analysis of environmental risk factors (Table 4) associated with *D. immitis* infection, univariate logistic regression showed that dogs acquired as adults, as well as stray or abandoned dogs, had a higher risk of beeing positive than dogs born to the current owner. Prolonged stay in Bosnia and Herzegovina was also associated with an increased risk of infection. In addition, sampling performed between 10:00 and 16:00 h was associated with a higher likelihood of a positive result. Dogs that had not been treated against endoparasites had a higher risk of infection, as did dogs without ectoparasite control. Dogs with previous therapy were more likely to test positive than dogs without previous therapy.

In the multivariate logistic regression analysis, a significantly increased risk of infection remained in dogs acquired as adults, as well as in dogs that had spent a longer period in BiH. Commercial diet was associated with a higher risk of infection compared to home-prepared food. Sampling between 10:00 and 16:00 h remained significantly associated with infection after model adjustment. A markedly increased risk was also observed in the multivariate model among dogs with previous therapy.

### 3.3. Risk Factor for D. repens Infection

*Individual risk factors*. In the analysis of individual factors (Table 5) associated with *Dirofilaria repens* infection, univariable logistic regression showed that underweight dogs had a higher risk of infection. In the multivariable logistic regression analysis, underweight dogs remained at higher risk of infection.

*Environmental risk factors*. In the analysis of environmental risk factors (Table 6) associated with *D. repens* infection, univariable logistic regression showed that dogs housed outdoors had a significantly higher risk of infection. Being positive was related with short trips within BiH and prolonged stay in BiH. Sampling performed between 10:00 and 16:00 h was associated with a higher likelihood of infection. Regarding the preventive measures, unvaccinated dogs and dogs vaccinated only against rabies had a higher risk of being positive, while also showed an increased risk. Higher risk of infection with *D. repens* was revealed in dogs which not treated against endoparasites as well as in dogs without ectoparasite control.

In the multivariable logistic regression analysis, dogs with short trips within BiH remained at significantly increased risk of infection after adjustment of the model. None of the other evaluated environmental risk factors retained statistical significance in the multivariable model.

### 3.4. Risk Factors for A. reconditum Infection

*Individual risk factors*. In the analysis of individual risk factors (Table S1) associated with *A. reconditum* infection, univariable logistic regression showed that neutered dogs had a significantly higher risk of infection compared to non-neutered dogs.

*Environmental risk factors*. In the analysis of environmental risk factors (Table S2) associated with *A. reconditum* infection, univariable logistic regression showed that dogs not treated against endoparasites had a significantly higher risk of infection. Unowned dogs also exhibited a significantly increased risk of infection compared to owned dogs. In the multivariable logistic regression analysis, dogs not treated against endoparasites remained at significantly higher risk of infection. Unowned dogs also retained a significantly increased risk of infection after adjustment.

### 3.5. Results of Diagnostic Methods for the Detection of Filarial Infections in Dogs

The diagnostic performance of the modified Knott test (mKnott), Heartworm antigen ELISA (HW agELISA), and PCR-based assays was evaluated in dogs infected with *D. immitis*, *D. repens*, and *A. reconditum* (Table 7).

Among the 45 dogs infected with *D. immitis*, the highest detection rate was achieved by species-specific PCR, which identified 37 cases (82.22%). The HW agELISA detected 28 infections (62.22%), whereas the modified Knott test identified only 16 positive samples (35.56%). For the 56 dogs infected with *D. repens*, species-specific PCR demonstrated the highest diagnostic sensitivity, detecting all infected animals (100%). In contrast, the modified Knott test detected 23 cases (41.07%). In the group of six dogs infected with *A. reconditum*, both pan-filarial and species-specific PCR successfully detected all infections (100%). The modified Knott test identified five of the six cases (83.33%).

Within the group of *D. immitis*-positive dogs (*N* = 45), pairwise method comparisons were performed to calculate McNemar’s *p*, matched odds ratio (OR), and Cohen’s kappa (κ) for agreement, based on the actual proportion of infections detected by each observed method. All other methods demonstrated a significantly higher detection rate compared to the modified Knott’s test (m-Knott): HW ag ELISA (*p* = 0.0075), pan-filarial PCR (*p* = 0.00022), and species-specific PCR (*p* = 0.000006). The differences between the HW ag ELISA and PCR methods, as well as between the two PCR methods, did not reach statistical significance. The highest agreement was found between the pan-filarial PCR and the species-specific PCR methods (Table S3). By excluding from the pairwise comparison only agELISA-positive dogs that had been treated with macrocyclic lactones in the year before sampling (six compared to the m-Knott test and five compared to the PCR tests), the difference between the m-Knott and HW ag ELISA did not reach statistical significance with poor to moderate agreement between the methods, and compared to the HW ag ELISA test, a significantly higher detection rate of the species-specific PCR was found, while the pan-filarial PCR also showed a higher chance of detection at the border of significance (Table S4).

### 3.6. Results of Phylogenetic Analysis

The genetic variations observed amongst the *D. immitis* samples analyzed in this study based on partial sequences of the 5.8S-*ITS2* genes ranged from 7.3% to 11.2%. The intra-species genetic distance between the sequences of *D. immitis* obtained in this study and analogous sequences available from GenBank ranged from 8.9% to 15.3%, with the closest match being to Portuguese isolates from mosquitoes (LN626266 and LN626267). Interspecies sequence distance between the *D. immitis* sequences from this study and *D. repens* sequences, both from this study (PQ615128–PQ615130) and GenBank (AY693808, MK942385, and JQ039743), ranged from 28.7% to 31.8%. BLAST analysis of the partial 5.8S-ITS2 gene sequences revealed that the *D. repens* sequences from this study shared 99.7–100% similarity among themselves and 96–100% similarity with matching sequences from GenBank. The highest levels of similarity were observed with a dog isolate from Russia (MN200338) and a human isolate from France (MK942385). At the same time, the most divergent sequence was a dog isolate from Sri Lanka (ON332069). Additionally, the *A. reconditum* isolate from this study exhibited the highest similarity (95.9%) to a Brazilian isolate (KX932126) and the greatest diversity (88.1%) from a Colombian isolate (MZ468151), both obtained from dogs.

**Figure 2:**
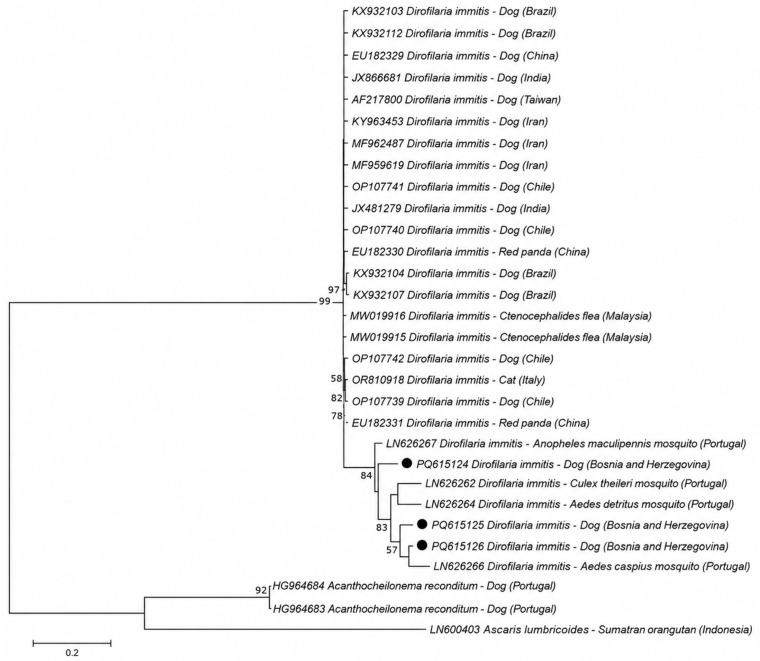
Phylogenetic tree of *D. immitis* isolates (indicated by ●) and related species.

The phylogenetic analysis based on the partial 5.8S-*ITS2* genes illustrated that all *D. immitis* isolates obtained in this study form a distinct clade, suggesting a localized transmission cycle of *D. immitis* in the region (Figure 2). These strains are closely related to mosquito strains from Portugal (LN626262, LN626264, and LN626266) and have a strong bootstrap value supporting the clade.

**Figure 3:**
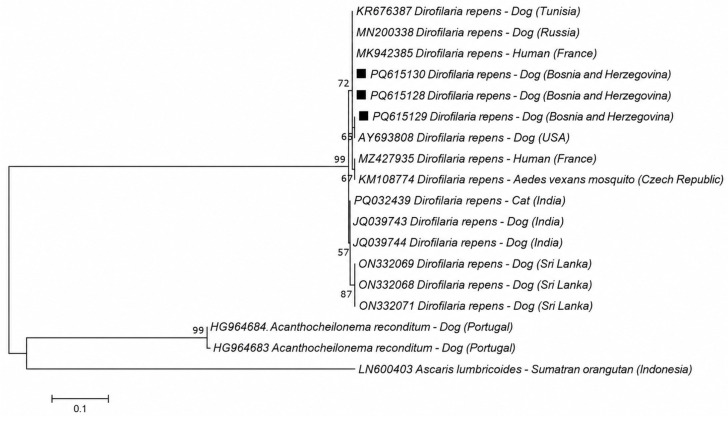
Phylogenetic tree of *D. repens* isolates (indicated by ■) and related species.

The BiH *D. repens* strains (PQ615128, PQ615129, and PQ615130) cluster together, revealing high genetic similarity, and are closely related to *D. repens* strains from Russia (MN200338), Tunisia (KR676387), USA (AY693808) and a human case in France (MK942385) (Figure 3).

The clustering of the *A. reconditum* isolate (PQ615127) with strains from Brazil (KX932126, KX932127) and Colombia (MZ468151) is supported by high bootstrap values (Figure S1). Contrarily, isolates from India (GU593976, GU593977) and Iran (JN819184) create separate clades, highlighting the genetic variation possibly associated with regional environmental factors. However, data in GenBank on *A. reconditum* sequences from Europe is scarce, making it challenging to determine how the studied Bosnian strain relates to other European strains.

## 4. Discussion

Epidemiology. This is the first geographically stratified study in which the zoonotic filarial species *D. immitis*, *D. repens*, and *A. reconditum* were molecularly confirmed and phylogenetically analyzed in the Republic of Srpska (BiH). The total prevalence of filarial infections was 21.29%, with *D. repens* detected in 13.40%, followed by *D. immitis* with 10.77%, and *A. reconditum* in 1.43% of dogs. These prevalences are higher than those reported in previous studies from this region [33], which may be explained by differences in the applied methodology, sampled population, and geographical coverage, rather than necessarily reflecting a true increase in prevalence. The expansion of *Dirofilaria* spp. has been associated with climate change, urbanization, and the transportation of dogs [12,34,35,36,37]. The highest prevalence rates were recorded in regions of RS characterized by large river systems, which is consistent with the vector-borne nature of dirofilariosis and comparable to findings reported from Romania [38]. The observed prevalence of *D. immitis* corresponds to values reported in Mediterranean and North African countries [39,40,41,42,43], and follows the expansion trend predicted by climate-based models [7,44,45].

The pronounced geographical heterogeneity in the distribution of *D. immitis* in RS, characterized by high-risk hotspots in the Gradiška region and low prevalence in other regions, reflects the typical pattern of spread observed in newly endemic countries. A similar model has been described in European countries, where, following the initial emergence of infection, localized high-risk microfoci gradually develop under the influence of dog movement, local ecological conditions, and prolongation of the mosquito season [12,44,46]. The spread of *D. immitis* in RS is occurring both horizontally, through the establishment of local hotspots, and vertically, toward higher altitudes, as a result of the synergistic effects of dog movement, global warming, and the expansion of competent vector species [47,48,49].

High prevalences of *D. repens* have been reported in the Mediterranean region of Europe [50] and in Southeast Asia [51], while in Africa this filarial species is endemic from the northern Mediterranean coast to subtropical regions [52]. The presence of competent vectors and the importation of dogs create epidemiological pressure for potential endemic establishment. In RS, no significant regional differences in the prevalence of *D. repens* were observed, indicating that the parasite has completed its adaptation across the entire investigated area.

This study provides the first data on the presence of *A. reconditum* in dogs in RS. The observed low prevalence (1.43%) is consistent with findings reported in Europe and neighbouring Balkan countries [21,53,54,55]. Higher prevalence rates have been reported in warmer southern European regions with denser dog populations, such as Italy and Spain [54,56,57,58]. The observed prevalence may be associated with the absence of preventive antiparasitic treatment, particularly among stray and unowned dogs. Overall, the prevalence of *A. reconditum* in RS remains substantially lower than that of *Dirofilaria* spp., indicating the comparatively limited epidemiological importance of this filarial species. Individual risk factors. No significant difference in the prevalence of filarial infections was observed between male and female dogs. On contrary, neutered dogs had higher odds of infection with *A. reconditum*. This was associated with their stray or shelter status, since positive dogs were originated from shelters where neutering is routinely performed. These findings are consistent with most studies, which do not recognize these variables as relevant biological predictors [54,59]. Hair coat length was significant for *D. immitis*, which supports the hypothesis that shorter hair increases exposure to mosquito bites [60]. This protective effect did not become significant for *D. repens*, probably due to their different spatial distribution, nor for *A. reconditum*, apparently due to the different biology of its vectors.

A significant difference in prevalence of *D. immitis* and *D. repens* was found between age groups, where the higher prevalence was found in older dogs as a consequence of cumulative exposure to vectors [61,62]. On the other side, younger dogs exhibited a higher risk for *A. reconditum* infection, probably due to more direct contact with ectoparasites in shelters [53,59]. Similar findings have been reported in other studies emphasizing that age has secondary importance compared with environmental factors [56].

Body condition identified thinness/malnutrition as the most consistent predictor for *D. immitis* and *D. repens*, suggesting that the host’s immune status and general health substantially influence the establishment and persistence of infection [1,63]. Furthermore, chronic diseases and nutritional status may modify susceptibility and receptivity to filarial infections. All dogs positive for *A. reconditum* were in good body condition, indicating its low pathogenic potential and weaker dependence on the host’s physiological status [54,59].

Mixed-breed dogs were found to have an increased risk of *D. immitis* infection, likely due to reduced owner supervision and greater exposure to vectors [61], whereas such genetic predisposition does not appear to play a major role in *D. repens* and *A. reconditum* infections [21,54,59]. Clinical signs were associated only with *D. immitis* infection, reflecting its pronounced pathogenicity [1,64].

Environmental risk factors. The obtained results confirm that the origin of the dog is an important epidemiological indicator of previous exposure and housing conditions, and therefore a significant factor in assessing the risk of infection with all three filarial species. In the case of *D. immitis*, acquisition of dogs older than one year emerged as an independent predictor of infection, which is consistent with findings from other studies [34,56] emphasizing the importance of previous exposure in different endemic areas.

Housing conditions, purpose, and ownership status of dogs demonstrated a common epidemiological pattern influencing the risk of filarial infections. Outdoor housing, shelters, working/hunting purposes, and stray status were associated with increased exposure to parasites, although with variable statistical stability. For *A. reconditum*, the strongest association was observed with stray status and shelter housing, which is consistent with facilitated transmission via ectoparasites [54,57,59]. In contrast, housing conditions were not identified as independent predictors for *D. repens* and *D. immitis*, despite their epidemiological relevance, likely because the risk of dirofilariosis is primarily determined by the spatial distribution of vectors and exposure to mosquitoes [53,65].

The travel history of dogs proved to be a significant environmental risk factor for all filarial species. For dirofilariosis, travel to other regions within the country represented an independently significant predictor of infection, as it enables exposure to different vector populations and infection in endemic hotspots [53,66]. Differences between species were reflected in the duration of exposure required for infection—shorter for *D. repens* and longer for *D. immitis*—which may indicate differences in their distribution and transmission intensity across various regions.

The factor of previous treatment emerged as an independent predictor only for *D. immitis*, with treated dogs showing a substantially higher risk of recurrent positivity. This finding may be explained by treatment failure, antigen persistence, and possible reinfection in endemic areas. Antigen tests may remain positive after treatment due to residual circulating antigens, potentially leading to an apparent reinfection [67,68], while the absence of preventive measures enables true reinfection [69]. These findings highlight the specific epidemiological relevance of treatment history in *D. immitis* infection, while also emphasizing the need for caution in interpretation because of potential diagnostic and biological limitations.

Different deworming practices, unless performed with macrocyclic lactones, are not a direct causal factor and do not influence the occurrence of microfilaremia, but they reflect the level of care of the owner, as shown by our results, which indicate that for *Dirofilaria* spp. this factor lost significance after adjustment. In *A. reconditum*, the absence of deworming was an independently significant factor, which may suggest that this variable is a marker of owner care and the overall level of veterinary care in combination with the dog’s exposure to vectors. However, the statistical analysis for this filariasis must certainly be interpreted with caution due to the small number of positive findings [53,54,65].

Commercial diet was associated with a higher risk of *D. immitis*. There is no proven reasonable biological mechanism for this association; however, it is evident that the highest prevalence was in dogs in shelters, where such a diet is also present, which in this statistical model resulted in this outcome. With regard to mosquito protection and ectoparasite treatment, differences were observed in accordance with the biology of the parasites. For *D. immitis* and *D. repens*, the absence of mosquito protection would be expected to increase infection risk; however, this was not statistically confirmed, indicating that infection depends on a complex interaction among the dog’s status, purpose, movement patterns, and climatic, spatial, and ecological factors that determine vector presence and activity [54,65,66]. In contrast, the absence of ectoparasite treatment had direct significance for *A. reconditum*, as transmission depends on the presence of fleas and lice within the dog’s immediate microenvironment [59,65].

Sampling time does not represent a true risk factor; however, it has been shown to influence diagnostic sensitivity due to the circadian variation of *D. immitis* microfilaremia [70,71]. A similar but less pronounced pattern has been described for *D. repens* [72]. In the present study, the period between 10:00 and 16:00 h emerged as an independently significant interval for *D. immitis*, partially significant for *D. repens*, and associated with a trend toward increased risk for *A. reconditum*. This deviation from the classical evening/nocturnal peaks may be explained by the use of multiple biomarkers (microfilariae, female antigens, and DNA) and by variations in the behavior of local vectors [54,71,72], which may contribute to alterations in the typical levels of microfilaremia within the newly established ecological niches of BiH. Although the findings related to sampling time may partly reflect the study design, they nevertheless suggest that this factor may have practical importance in optimizing diagnostic protocols.

An indirect effect was observed for *D. repens*, where lack of vaccination or vaccination only against rabies was associated with a higher risk in univariable analysis, further emphasizing the importance of veterinary care as an indirect marker of infection risk [53,54,65].

Laboratory diagnostics of filarial infections differ substantially in terms of sensitivity and applicability. While serological antigen tests represent the standard diagnostic approach for *D. immitis*, the diagnosis of *D. repens* and *A. reconditum* still relies primarily on microscopic and molecular methods. Due to nonspecific clinical signs and the absence of serological assays, detection of these species in clinical practice is largely based on the differentiation of microfilariae during routine testing of dogs for *D. immitis* [1,24,66].

In the present study, species-specific PCR, pan-filarial PCR, HW agELISA, and the modified Knott’s test demonstrated a descending order of diagnostic sensitivity, which is consistent with reports highlighting the high sensitivity of molecular methods for the differentiation of canine filarial species [64,73,74,75]. This advantage is particularly important in cases of low microfilaremia, subclinical infections, and diagnostically ambiguous cases, in which classical methods may show limited sensitivity [22,66]. The relatively low sensitivity of the modified Knott’s test observed in this study is consistent with the known limitations of methods dependent on the presence of microfilariae in peripheral blood. Antigen ELISA tests for *D. immitis* exhibit good, but incomplete, sensitivity, particularly in cases of low parasite burden and limited availability of circulating antigens [63,76,77,78,79,80].

Differences in diagnostic performance among methods arise from the detection of distinct biological targets of infection—microfilariae, circulating female antigens, or parasite DNA—which directly influences comparative sensitivity and clinical applicability [1,22,63]. Changes in microfilaremic status, particularly following previous treatment, may reduce the likelihood of detecting microfilariae and parasite DNA, whereas antigen tests may remain positive. Therefore, interpretation of diagnostic results should consider treatment history, and combined testing for both antigen and microfilariae is recommended [69,76]. The results of the comparison of diagnostic tests in the present study confirm the lower sensitivity of microscopic and specific PCR approaches for *D. immitis*, when dogs treated with macrocyclic lactones were included in the study.

The combined use of diagnostic methods is considered the most reliable approach, as it enables the detection of different stages of infection and reduces the risk of false-negative results [63,76]. The findings of this study support an integrated diagnostic algorithm combining PCR, serological, and microscopic methods to achieve more reliable diagnosis of canine filarial infections [69]. The small number of sequenced samples limits phylogenetic analysis. *D. immitis* isolates show marked genetic diversity with distant regions of South America and Asia and closeness to the European isolate from Portugal. *D. repens* isolates cluster with isolates from the Mediterranean (Tunisia) and European isolates (Russia), which may indicate a broad geographic genetic stability of this isolate, while the closeness to French isolates from humans suggests a possible zoonotic potential. The closeness of the BiH isolates of *A. reconditum* to isolates from Brazil and South America can be explained by the small number of sequenced samples from Europe and the lack of comparable data.

## 5. Conclusions

The results of this study confirm the substantial distribution of canine filarial infections in the RS, with an overall prevalence of 21.29%. The highest frequency was recorded for *D. repens* (13.40%), followed by *D. immitis*, while *A. reconditum* was detected sporadically, indicating the considerable veterinary and public health importance of these parasites. Significant regional differences in the prevalence of filarial infections were observed, with the highest frequency recorded in dogs from the northwestern part of the RS, particularly the Gradiška and Prijedor regions, and in the zone characterized by a temperate continental climate. Risk factor analysis showed that the occurrence of infection was influenced by both individual dog-related characteristics, including body condition, breed status, and coat length, and environmental factors, such as travel history, dog origin, previous treatment, and inadequate antiparasitic protection. The marked genetic diversity of *D. immitis* isolates, the presence of a local transmission cycle in the region, and their close relationship with strains from Portugal may indicate common or similar routes of spread. The high genetic similarity of BiH *D. repens* isolates to zoonotic strains from Europe and other parts of the world confirms their zoonotic potential, whereas the observed differences among geographically distant isolates suggest the influence of regional factors on the genetic variability of these parasites. The obtained results emphasize the need for continuous epidemiological surveillance, regular implementation of preventive measures, systematic vector control, and owner education, with the aim of reducing the risk of filarial spread and potential zoonotic transmission of these nematodes.

## Figures and Tables

**Table 1:** Primers used for PCR detection and identification of filarial species [30].

Family/Species	Target Gene	Primers	Sequence (5′–3′)
Onchocercidae (panfilarial)	rDNA *ITS*	DIDR-F1	AGT GCG AAT TGC AGA CGC ATT GAG
		DIDR-R1	AGC GGG TAA TCA CGA CTG AGT TGA
*Dirofilaria immitis*	*COI*	DI COI-F1	AGT GTA GAG GGT CAG CCT GAG TTA
		DI COI-R1	ACA GGC ACT GAC AAT ACC AAT
*Dirofilaria repens*	*COI*	DR COI-F1	AGT GTT GAT GGT CAA CCT GAA TTA
		DR COI-R1	GCC AAA ACA GGA ACA GAT AAA ACT
*Acanthocheilonema reconditum*	*ITS2*	A.rec-F1	CAG GTG ATG GTT TGA TGT GC
		A.rec-R1	CAC TCG CAC TGC TTC ACT TC

*COI*-cytochrome oxidase subunit 1; *ITS*—Internal Transcribed Spacer.

**Table 2:** Prevalence of *D. immitis*, *D. repens* and *A. reconditum* in regions of Republika Srpska.

Filarial Nematodes	Banja Luka (*N* = 68)	Prijedor (*N* = 50)	Gradiška (*N* = 82)	Doboj (*N* = 56)	Bijeljina (*N* = 61)	Istocno Sarajevo *(N* = 46)	Trebinje *(N* = 55)	Total (*N* = 418)	χ^2^	*p*
				*n* (%)						
DI	4 (5.9)	0	21 (25.6)	9 (16.1)	5 (8.2)	1 (2.2)	5 (9.1)	45 (10.8)	32.3	***
DR	3 (4.4)	12 (24)	14 (17.1)	7 (12.5)	8 (13.1)	5 (10.9)	7 (12.7)	56 (13.4)	10.9	0.09
AR	0	0	5 (6.1)	0	0	0	1 (1.8)	6 (1.4)	16.8	*
DI-DR	1 (1.5)	0	6 (7.3)	3 (5.4)	3 (4.9)	0	1 (1.8)	14 (3.4)	9.7	0.14
DI-DR-AR	0	0	1 (1.2)	0	0	0	1 (1.8)	2 (0.5)	4.4	0.62

*N*—number of tested dogs; *n*—number of positive dogs; * = *p* < 0.05; *** = *p* < 0.001; DI—*Dirofilaria immitis*; DR—*Dirofilaria repens*; AR—*Acanthocheilonema reconditum*.

**Table 3:** Univariable and multivariable logistic regression analysis of individual risk factors associated with *Dirofilaria immitis* infection in dogs.

Risk Factor	Tested Dogs (*N* = 418)	Positive Dogs (*N* = 45)	Prevalence % (95% CI)	Univariable Analysis OR (95% CI)	Multivariable Analysis aOR (95% CI)
**Sex**					
Female (Ref.)	206	22	10.7% (6.8–15.7)	1	-
Male	212	23	10.8% (7.0–15.9)	1.0 (0.6–1.9)	-
**Neutered status**					
No (Ref.)	280	29	10.4% (7.1–14.6)	1	-
Yes	138	16	11.6% (6.8–18.1)	1.5 (0.6–2.8)	-
**Coat length**					
Long (Ref.)	66	3	4.5% (0.9–12.7)	1	1
Short	262	31	11.8% (8.2–16.2)	2.8 (0.8–9.5)	3.8 (1.0–13.6) *
Medium	90	11	12.2% (6.3–20.6)	2.9 (0.8–10.9)	3.4 (0.9–13.5)
**Age (years)**					
≤2 (Ref.)	96	7	7.3% (3.0–14.5)	1	1
>2 to 8	286	32	11.2% (7.8–15.4)	1.6 (0.7–3.8)	1.7 (0.7–4.0)
>8	36	6	16.7% (6.4–32.8)	2.5 (0.8–8.2)	2.3 (0.7–8.2)
**Body weight (kg)**					
>40 (Ref.)	30	2	6.7% (0.8–22.1)	1	-
≤5	25	3	12.0% (2.5–31.2)	1.9 (0.3–12.4)	-
>5 to 20	249	25	10.0% (6.6–14.4)	1. 6 (0.4–7.0)	-
>20 to 40	114	15	13.2% (7.6–20.8)	2.1 (0.5–9.8)	-
**Body condition**					
Working (Ref.)	51	1	2.0% (0.05–10.4)	1	1
Breeding	30	6	20.0% (7.7–38.6)	12.5 (1.4–109.7) *	11.4 (1.3–102.7) *
Good	294	30	10.2% (7.0–14.3)	5.7 (0.8–42.6)	5.0 (0.6–38.5)
Obese	32	3	9.4% (2.0–25.0)	5.2 (0.5–52.1)	3.6 (0.3–39.2)
Thin (underweight)	11	5	45.5% (16.7–76.6)	41.7 (4.1–419.0) **	26.9 (2.2–327.4) **
**Breed status**					
Purebred (Ref.)	241	18	7.5% (4.5–11.6)	1	1
Mixed-breed	177	27	15.3% (10.3–21.6)	2.2 (1.2–4.2) *	2.2 (1.1–4.4) *
**Clinical symptoms**					
No (Ref.)	393	39	9.9% (7.2–13.3)	1	1
Yes	25	6	24.0% (9.4–45.1)	2.9 (1.1–7.6) *	1.7 (0.5–5.9)

* *p* < 0.05; ** *p* < 0.01; Prevalence 95% CI calculated using Clopper–Pearson exact method.

**Table 4:** Analysis of environmental risk factors associated with *Dirofilaria immitis* infection in dogs (Univariable and multivariable logistic regression).

Risk Factor	Tested Dogs (N = 418)	Positive Dogs (N = 45)	Prevalence % (95% CI)	Univariable Analysis OR (95% CI)	Multivariable Analysis aOR (95% CI)
**Purpose of dog**					
Pet (indoor) (Ref.)	88	6	6.8% (2.5–14.3)	1	—
Pet (outdoor)	97	14	14.4% (8.1–23.0)	2.3 (0.8–6.3)	—
Hunting	130	13	10.0% (5.4–16.5)	1.78 (0.6–4.2)	—
Working	103	12	11.7% (6.2–19.5)	2.11 (0.7–5.0)	—
**Housing conditions**					
Indoors (alone) (Ref.)	36	2	5.6% (0.7–18.7)	1	1
Outdoor (constant)	234	25	10.7% (7.0–15.4)	2.3 (0.5–9.0)	6.4 (0.4–95.7)
Indoors (owner)	63	5	7.9% (2.6–17.6)	1.5 (0.3–8.0)	2.2 (0.1–43.7)
Shelter dog	85	13	15.3% (8.4–24.7)	3.1 (0.7–14.4)	8.7 (0.4–181.2)
**Origin of dog**					
Born at owner (Ref.)	64	3	4.7% (1.0–13.0)	1	1
Puppy < 3 months	173	13	7.5% (4.1–12.4)	1.7 (0.5–6.0)	2.6 (0.6–10.5)
Up to 1 year	28	3	10.7% (2.3–28.2)	2.4 (0.5–12.9)	1.5 (0.2–10.8)
Adult > 1 year	59	10	16.9% (8.4–29.0)	4.2 (1.1–15.9) *	5.8 (1.2–29.4) *
Stray/Abandoned	94	16	17.0% (10.1–26.0)	4.2 (1.2–15.0) *	6.5 (0.6–68.7)
**Travel history**					
Short trips in BiH (Ref.)	30	2	6.7% (0.8–22.1)	1	1
Unknown	101	11	10.9% (5.6–18.7)	1.3 (0.3–5.7)	1.3 (0.3–4.9)
No travel	220	18	8.2% (4.9–12.7)	1.7 (0.4–8.2)	1.3 (0.2–10.0)
Long stay in BiH	24	9	37.5% (18.8–59.4)	8.4 (1.6–44.0) *	4.5 (1.2–16.8) *
Abroad	43	5	11.6% (3.9–24.6)	1.8 (0.3–1.2)	2.7 (0.5–15.6)
**Mosquito protection**					
Chemical (Ref.)	24	1	4.2% (0.1–21.1)	1	—
Unprotected	363	41	11.3% (8.2–15.1)	2.9 (0.4–22.3)	—
Nets	31	3	9.7% (2.0–25.8)	2.5 (0.2–25.3)	—
**Feeding type**					
Owner-prepared (Ref.)	50	2	4.0% (0.5–13.7)	1	1
Commercial feed	287	37	12.9% (9.3–17.3)	3.6 (0.8–15.2)	4.0 (1.3–12.4) *
Mixed feed	81	6	7.4% (2.8–15.4)	1.9 (0.4–9.9)	1.2 (0.2–8.2)
**Sampling time**					
After 16 h (Ref.)	62	2	3.2% (0.4–11.2)	1	1
07–10 h	128	13	10.2% (5.6–16.7)	3.4 (0.7–15.5)	2.9 (0.6–15.0)
10–16 h	228	30	13.2% (9.1–18.3)	4.6 (1.1–19.6) *	5.0 (1.1–23.6) *
**Vaccination status**					
Rabies only (Ref.)	205	17	8.3% (4.9–13.0)	1	1
Unvaccinated	44	7	15.9% (6.7–30.1)	2.09 (0.8–5.4)	2.3 (0.7–8.3)
Rabies + DHPPL	169	21	12.4% (7.9–18.3)	1.57 (0.8–3.1)	2.4 (1.0–6.1)
**Deworming**					
Treated (Ref.)	275	23	8.4% (5.4–12.3)	1	1
Untreated	143	22	15.4% (9.9–22.4)	2.0 (1.1–3.7) *	2.3 (0.9–5.7)
**Ectoparasites present**					
No (Ref.)	377	39	10.3% (7.5–13.8)	1	—
Yes	41	6	14.6% (5.6–29.2)	1.5 (0.6–3.8)	—
**Ectoparasite control**					
Treated (Ref.)	153	10	6.5% (3.2–11.6)	1	1
Untreated	265	35	13.2% (9.4–17.8)	2.2 (1.1–4.5) *	1.58 (0.6–4.5)
**Ownership status**					
Owned (Ref.)	307	28	9.1% (6.2–12.9)	1	1
Unowned	111	17	15.3% (9.2–23.3)	1.8 (0.9–3.5)	4.60 (0.6–34.3)
**Previous therapy**					
No (Ref.)	406	39	9.6% (6.9–12.9)	1	1
Yes	12	6	50.0% (21.1–78.9)	9.4 (2.9–30.6) ***	10.53 (2.3–49.4) **

* *p* < 0.05; ** *p* < 0.01; *** *p* < 0.001; Prevalence 95% CI calculated using Clopper–Pearson exact method.

**Table 5:** Analysis of individual factors associated with *Dirofilaria repens* infection in dogs (Univariable and multivariable logistic regression).

Risk Factor	Tested Dogs (*N* = 418)	Positive Dogs (*N* = 56)	Prevalence % (95% CI)	Univariable Analysis OR (95% CI)	Multivariable Analysis aOR (95% CI)
**Sex**					
Female (Ref.)	212	28	13.2 (8.9–18.6)	1	—
Male	206	28	13.6 (9.3–19.1)	1.0 (0.6–1.8)	—
**Neutered status**					
Yes (Ref.)	138	16	11.6 (6.8–18.1)	1	—
No	280	40	14.3 (10.5–19.0)	1.3 (0.7–2.3)	—
**Coat length**					
Short (Ref.)	262	31	11.8 (8.2–16.3)	1	1
Medium	90	16	17.8 (10.4–27.0)	1.6 (0.8–3.1)	1.6 (0.8–3.3)
Long	66	9	13.6 (6.4–24.2)	1.2 (0.5–2.6)	1.1 (0.5–2.7)
**Age (years)**					
≤2 years (Ref.)	96	8	8.3 (3.7–15.7)	1	1
>2 to 8 years	286	41	14.3 (10.5–19.0)	1.8 (0.8–4.1)	1.8 (0.8–4.0)
>8 years	36	7	19.4 (8.2–36.0)	2.7 (0.9–8.0)	2.7 (0.9–8.4)
**Body weight (kg)**					
≤5 kg (Ref.)	25	1	4.0 (0.1–20.4)	1	1
>5–20 kg	249	35	14.1 (10.1–18.9)	3.9 (0.5–30.0)	3.8 (0.5–30.7)
>20–40 kg	114	15	13.2 (7.6–20.8)	3.6 (0.5–28.9)	3.5 (0.4–29.2)
≥40 kg	30	5	16.7 (5.6–34.7)	4.8 (0.5–44.2)	8.1 (0.8–79.0)
**Body condition**					
Breeding (Ref.)	30	2	6.7 (0.8–22.1)	1	1
Working	51	9	17.6 (8.4–30.9)	3.0 (0.6–14.9)	3.8 (0.7–21.2)
Good	294	36	12.2 (8.7–16.5)	1.2 (0.5–8.6)	2.1 (0.4–10.0)
Obese	32	4	12.5 (3.5–29.0)	2.0 (0.3–11.8)	1.8 (0.3–11.5)
Thin (underweight)	11	5	45.5 (16.7–76.6)	11.7 (1.8–75.1) *	12.2 (1.7–90.2) *
**Breed status**					
Purebred (Ref.)	241	28	11.6 (7.9–16.3)	1	1
Mixed breed	177	28	15.8 (10.7–22.1)	1.4 (0.8–2.5)	1.4 (0.7–2.8)
**Clinical symptoms**					
No (Ref.)	393	52	13.2 (10.1–17.0)	1	—
Yes	25	4	16.0 (4.5–36.1)	1.3 (0.4–3.8)	—

* *p* < 0.05; Prevalence 95% CI calculated using Clopper–Pearson exact method.

**Table 6:** Analysis of environmental risk factors associated with *Dirofilaria repens* infection in dogs (univariable and multivariable logistic regression).

Risk Factor	Tested Dogs (*N* = 418)	Positive Dogs (*N* = 56)	Prevalence % (95% CI)	Univariable Analysis OR (95% CI)	Multivariable Analysis aOR (95% CI)
**Purpose of dog**					
Pet (indoor)	88	7	8.0 (3.3–15.7)	1	1
Pet (outdoor)	97	16	16.5 (9.8–25.2)	2.3 (0.9–5.9)	1.8 (0.4–7.9)
Hunting	130	18	13.8 (8.4–20.9)	1.9 (0.7–4.7)	1.2 (0.3–5.4)
Working	103	15	14.6 (8.4–23.0)	2.0 (0.8–5.1)	1.8 (0.4–8.5)
**Housing conditions**					
Indoors (owner) (Ref.)	63	3	4.8 (1.0–13.3)	1	1
Indoors (alone)	36	5	13.9 (4.7–29.5)	3.23 (0.7–14.2)	1.8 (0.1–39.2)
Outdoor (constant)	234	39	16.7 (12.2–22.1)	4.00 (1.2–13.5) *	2.7 (0.4–17.6)
Shelter dog	85	9	10.6 (5.0–19.2)	2.37 (0.6–8.8)	1.1 (0.2–7.5)
**Origin of dog**					
Born at owner (Ref.)	64	6	9.4 (3.5–19.3)	1	1
Puppy < 3 months	173	25	14.5 (9.6–20.7)	1.6 (0.6–4.2)	2.0 (0.7–6.0)
Up to 1 year	28	7	25.0 (10.7–44.9)	3.2 (1.0–10.7)	3.4 (0.9–13.4)
Adult > 1 year	59	6	10.2 (3.8–20.8)	1.1 (0.3–3.6)	1.6 (0.4–6.6)
Stray/Abandoned	94	12	12.8 (6.8–21.2)	1.4 (0.5–4.0)	6.7 (1.0–45.5)
**Travel history**					
Abroad (Ref.)	43	2	4.7 (0.6–15.8)	1	1
Unknown	101	8	7.9 (3.5–15.0)	1.8 (0.4–8.7)	1.6 (0.2–12.3)
No travel	220	33	15.0 (10.5–20.6)	3.6 (0.8–15.7)	2.5 (0.7–9.1)
Short trips in BiH	30	7	23.3 (9.9–42.3)	6.2 (1.2–32.6) *	4.0 (1.0–15.5) *
Long stay in BiH	24	6	25.0 (9.8–46.7)	6.8 (1.3–37.2) *	5.0 (0.9–29.0)
**Mosquito protection**					
Chemical (Ref.)	24	1	4.2 (0.1–21.1)	1	1
Unprotected—No	363	52	14.3 (10.8–18.4)	3.9 (0.5–29.1)	8.0 (0.4–155.1)
Nets	31	3	9.7 (2.0–25.8)	2.5 (0.2–25.3)	2.3 (0.3–20.3)
**Feeding type**					
Mixed feed (Ref.)	81	7	8.6 (3.5–16.8)	1	1
Owner prepared	50	7	14.0 (5.8–26.7)	1.7 (0.6–5.2)	1.4 (0.5–3.6)
Commercial feed	287	42	14.6 (10.7–19.4)	1.8 (0.8–4.2)	1.4 (0.4–5.3)
**Sampling time**					
After 16 h (Ref.)	62	4	6.5 (1.8–15.7)	1	1
7–10 h	128	11	8.6 (4.4–14.9)	1.4 (0.4–4.5)	1.0 (0.3–4.0)
10–16 h	228	41	18.0 (13.2–23.7)	3.2 (1.1–9.3) *	2.2 (0.7–7.1)
**Vaccination status**					
Rabies + DHPPL (Ref.)	169	14	8.3 (4.6–13.5)	1	1
Unvaccinated	44	9	20.5 (10.0–36.0)	3.0 (1.2–7.3) *	1.7 (0.5–6.0)
Rabies only	205	33	16.1 (11.3–21.8)	2.1 (1.1–4.1) *	1.9 (0.8–4.6)
**Deworming**					
Treated (Ref.)	275	29	10.5% (7.2–14.6)	1	1
Untreated	143	27	18.9% (12.9–26.2)	2.0 (1.1–3.5) *	1.0 (0.5–2.3)
**Ectoparasites present**					
No (Ref.)	377	47	12.5% (9.3–16.4)	1	1
Yes	41	9	22.0 (10.6–37.6)	2.0 (0.9–4.4)	1.6 (0.5–4.6)
**Ectoparasite control**					
Treated (Ref.)	153	12	7.8 (4.1–13.2)	1	1
Untreated	265	44	16.6 (12.4–21.6)	2.3 (1.2–4.6) *	2.0 (0.8–5.4)
**Ownership status**					
Stray/Unowned (Ref.)	111	11	9.9 (5.0–17.0)	1	1
Owned	307	45	14.7 (10.9–19.2)	1.6 (0.8–3.1)	4.5 (0.4–55.6)
**Previous therapy**					
Yes (Ref.)	12	0	0.0 (0.0–26.5)	1	—
No	406	56	13.8 (10.6–17.6)	4.0 (0.2–67.6)	—

* *p* < 0.05; Prevalence 95% CI calculated using Clopper–Pearson exact method.

**Table 7:** Successful of methods in diagnostic of filarial infections.

		mKnott	HW agELISA	PCR DIDR	PCR DI	PCR DR	PCR AR
	*N*	*n* (%)					
DI	45	16 (35.56)	28 (62.22)	33 (73.33)	37 (82.22)	/	/
DR	56	23 (41.07)	/	51 (91.07)	/	56 (100)	/
AR	6	5 (83.33)	/	6 (100)	/	/	6 (100)

*N*—number of total positive dogs; *n*—number of positive dogs; DI—*Dirofilaria immitis*; DR—*Dirofilaria repens*; AR—*Acanthocheilonema reconditum*.

## Data Availability

The data presented in this study are available on request from the corresponding author. The data are not publicly available because the dataset contains detailed individual-level information, and restricted access is required to maintain confidentiality.

## Supplementary Materials

The following supporting information can be downloaded at: https://www.mdpi.com/article/10.3390/ani16152361/s1, Table S1: Analysis of individual factors associated with *Acanthocheilonema reconditum* infection in dogs (Univariable and multivariable logistic regression); Table S2: Analysis of environmental risk factors associated with *Acanthocheilonema reconditum* infection in dogs (Univariable and multivariable logistic regression); Table S3: Comparative statistical analysis of the tests used, comparison of methods; Table S4: Comparative statistical analysis of the tests used without “agELISA only” positive previously ML treated dogs; Figure S1: Phylogenetic tree of *Acanthocheilonema reconditum* isolates (indicated by ▲) and related species.
