# Supplementary material for: Epidemiological and Molecular Investigation of Dirofilaria repens, Dirofilaria immitis, and Acanthocheilonema reconditum in Dogs in the Republic of Srpska (Bosnia and Herzegovina)
Source: Animals (Basel). 2026 Aug 2;16(15):2361. doi: 10.3390/ani16152361 (PMC13463490; doi:10.3390/ani16152361)
Supplement: Supplementary file 1 [file animals-16-02361-s001.zip › animals-4409352-supplementary.pdf]

Table S1. Analysis of individual factors associated with *Acanthocheilonema reconditum* infection in dogs (Univariable and multivariable logistic regression).

Risk factor	Tested dogs (N=418)	Positive dogs (N=6)	Prevalence % (95 % CI)	Univariable analysis OR (95% CI)	Multivariable analysis aOR (95% CI)
**Sex**					
Female (Ref.)	212	2	0.9 (0.1–3.4)	1	—
Male	206	4	1.9 (0.5–4.8)	2.079 (0.4-11.5)	—
**Neutered status**					
Yes (Ref.)	280	1	0.4 (0.01–2.0)	1	1
No	138	5	3.6 (1.2–8.2)	10.489 (1.2-90.7)*	6.4 (0.5-76.4)
**Coat length**					
Short (Ref.)	90	1	1.1 (0.03–6.0)	1	—
Medium	262	4	1.5 (0.4–3.9)	1.4 (0.2-12.5)	—
Long	66	1	1.5 (0.04–8.1)	1.4 (0.1-22.3)	—
**Age (years)**					
≤ 2 years (Ref.)	36	0	0.0 (0.0–9.7)	1	—
> 2 to 8 years	96	3	3.1 (0.6–8.9)	2.7 (0.14–53.7)	—
> 8 years	286	3	1.0 (0.2–3.0)	0.9 (0.05–17.7)	—
**Body weight (kg)**					
≤ 5 kg (Ref.)	30	0	0.0 (0.0–11.6)	1	—
> 5–20 kg	25	1	4.0 (0.1–20.4)	3.7 (0.15–93.4)	—
> 20–40 kg	249	2	0.8 (0.1–2.9)	0.6 (0.03–12.4)	—
≥ 40 kg	114	3	2.6 (0.5–7.5)	1.9 (0.09–39.7)	—
**Body condition**					
Breeding (Ref.)	30	0	0.0 (0.0–11.6)	—	—
Working	51	0	0.0 (0.0–7.0)	—	—
Good	294	6	2.0 (0.8–4.4)	—	—
Obese	32	0	0.0 (0.0–10.9)	—	—
Thin (underweight)	11	0	0.0 (0.0–10.9)	—	—
**Breed status**					
Purebred (Ref.)	241	1	0.4 (0.01–2.3)	1	1
Mixed breed	177	5	2.8 (0.9–6.5)	7.0 (0.8-60.2)	2.465 (0.2-29.6)
**Clinical symptoms**					
No (Ref.)	25	0	0.0 (0.0–13.7)	1	—
Yes	393	6	1.5 (0.6–3.3)	0.9 (0.1–14.8)	—

* $p < 0.05$; Prevalence 95% CI calculated using Clopper–Pearson exact method.

Table S2. Analysis of environmental risk factors associated with *Acanthocheilonema reconditum* infection in dogs (Univariable and multivariable logistic regression).

Risk factor	Tested dogs (N=418)	Positive dogs (N=6)	Prevalence % (95 % CI)	Univariable analysis OR (95% CI)	Multivariable analysis aOR (95% CI)
**Purpose of dog**					
Pet (indoor)	88	0	0.0 (0.0–4.2)	1	—
Pet (outdoor)	97	2	2.5 (0.3–8.6)	5.5 (0.3–116.4)	—
Hunting	130	1	0.8 (0.0–4.2)	2.0 (0.1–49.5)	—
Working	103	3	2.9 (0.6–8.3)	2.0 (0.1–49.5)	—
**Housing conditions**					
Indoors (owner) (Ref.)	63	0	0.0 (0.0–5.7)	1	—
Indoors (alone)	36	0	0.0 (0.0–9.7)	—	—
Outdoor (constant)	234	1	0.4 (0.01–2.4)	0.8 (0.03–20.5)	—
Shelter dog	85	5	5.9 (1.9–13.2)	8.2 (0.5–151.5)	—
**Origin of dog**					
Born at owner (Ref.)	64	0	0.0 (0.0–5.6)	1	—
Puppy < 3 months	173	1	0.6 (0.01–3.2)	1.1 (0.1–27.9)	—
Up to 1 year	28	0	0.0 (0.0–12.3)	—	—
Adult > 1 year	59	0	0.0 (0.0–6.1)	—	—
Stray / Abandoned	94	5	5.3 (1.7–11.9)	7.5 (0.4–139.8)	—
**Travel history**					
Abroad (Ref.)	220	0	0.0 (0.0–1.7)	1	—
Unknown	101	4	4.0 (1.1–9.8)	19.6 (1.1–350.4)	—
No travel	30	1	3.3 (0.1–17.2)	12.0 (0.5–287.4)	—
Short trips in BiH	24	1	4.2 (0.1–21.1)	15.0 (0.6–361.3)	—
Long stay in BiH	43	0	0.0 (0.0–8.2)	1.0 (0.02–52.3)	—
**Mosquito protection**					
Chemical (Ref.)	31	0	0.0 (0.0–11.2)	1	—
Unprotected - No	24	0	0.0 (0.0–14.2)	1.0 (0.1–49.7)	—
Nets	363	6	1.7 (0.6–3.7)	1.2 (0.1–21.7)	—
**Feeding type**					
Mixed feed (Ref.)	50	0	0.0 (0.0–7.1)	1	—
Owner prepared	287	5	1.7 (0.6–4.0)	2.0 (0.1–36.1)	—
Commercial feed	81	1	1.2 (0.03–6.7)	1.2 (0.1–29.3)	—
**Sampling time**					
After 16 h (Ref.)	128	0	0.0 (0.0–2.9)	1	—
07–10 h	228	6	2.6 (1.0–5.5)	7.36 (0.4–132.7)	—
10–16 h	62	0	0.0 (0.0–5.8)	1.0 (0.1–50.3)	—
**Vaccination status**					
Rabies + DHPPL (Ref.)	44	0	0.0 (0.0–8.2)	1	—
Unvaccinated	205	4	1.9 (0.5–4.9)	1.9 (0.1–34.6)	—
Rabies only	169	2	1.2 (0.1–4.2)	1.1 (0.1–22.4)	—
**Deworming**					
Treated (Ref.)	275	1	0.4 (0.01–2.0)	1	1
Untreated	143	5	3.5 (1.1–8.0)	9.9 (1.2-85.8)*	9.2 (1.1-80.9)*
**Ectoparasites present**					
No (Ref.)	41	0	0.0 (0.0–8.6)	1	—

Yes	377	6	1.6 (0.6–3.5)	1.4 (0.1–25.7)	—
**Ectoparasite control**					
Treated (Ref.)	153	0	0.0 (0.0–2.4)	1	—
Untreated	265	6	2.3 (0.8–4.9)	7.5 (0.4–134.9)	—
**Ownership status**					
Stray / Unowned (Ref.)	307	1	0.3 (0.01–1.8)	1	1
Owned	111	5	4.5 (1.5–10.2)	14.4 (1.7-124.9)*	13.6 (1.6-118.6)*
**Previous therapy**					
Yes (Ref.)	12	0	0.0 (0.0–26.5)	1	—
No	406	6	1.5 (0.6–3.2)	0.1–7.4)	—

* $p < 0.05$; Prevalence 95% CI calculated using Clopper–Pearson exact method.

Table S3: Comparative statistical analysis of the tests used, comparison of methods.

Comparison<br>N+ = 45	+ vs +<br>(+% vs +%)	Discordant<br>(+/− vs −/+)	McNemar p	OR (95% CI)	κ
m-Knott vs<br>HW ag ELISA	16 vs 28<br>(35.6% vs 62.2%)	3 vs 15	0.0075	5.0 (1.4–27.0)	0.253
m-Knott vs<br>PCR DIDR	16 vs 33<br>(35.6% vs 73.3%)	2 vs 19	0.00022	9.5 (2.3–84.1)	0.178
m-Knott vs<br>PCR DI COI	16 vs 37<br>(35.6% vs 82.2%)	1 vs 22	0.000006	22.0 (3.6–907.9)	0.138
HW ag ELISA vs<br>PCR DIDR	28 vs 33<br>(62.2% vs 73.3%)	11 vs 16	0.4421	1.5 (0.6–3.5)	−0.355
HW ag ELISA vs<br>PCR DI COI	28 vs 37<br>(62.2% vs 82.2%)	8 vs 17	0.1078	2.1 (0.9–5.7)	−0.319
PCR DIDR vs<br>PCR DI COI	33 vs 37<br>(73.3% vs 82.2%)	1 vs 5	0.2188	5.0 (0.6–236.5)	0.619

DI = Dirofilaria immitis; m-Knott = modified Knott test; HW ag ELISA = heartworm antigen ELISA; PCR = polymerase chain reaction; DIDR = panfilarial primers; N+ = number of total positive dogs; + = positive by the observed method; +% = ratio of the number of infections detected by the observed method to the number of total positive dogs; $p<0.05$ = significance level.

Note on calculation*: McNemar p is calculated using the exact two-sided test based on mismatched pairs. OR represents the matched odds ratio for the second method compared to the first, i.e. the ratio of the number of findings −/+ to +/−. The 95% CI for the OR is the exact conditional confidence interval, appropriate due to the small number of mismatched pairs in some comparisons.*

Table S4: Comparative statistical analysis of the tests used without "agELISA only" positive previously ML treated dogs.

**Comparison**<br>**N+ = 39**	**+ vs +**<br>**(+% vs +%)**	**Discordant**<br>**(+/− vs −/+)**	**McNemar**<br>***p***	**OR (95% CI)**	**κ**
m-Knott vs<br>HW ag ELISA	16 vs 22<br>(41.03% vs 58.97%)	3 vs 9	0.146	3.00 (0.81–11.08)	0.398
**N+ = 40**					
HW ag ELISA vs<br>PCR DIDR	23 vs 33<br>(57.5% vs 82.5%)	6 vs 16	0.052	2.7 (1.1–6.8)	−0.219
HW ag ELISA vs<br>PCR DI COI	23 vs 37<br>(57.5% vs 92.5%)	3 vs 17	0.003	5.7 (1.7–19.3)	−0.146

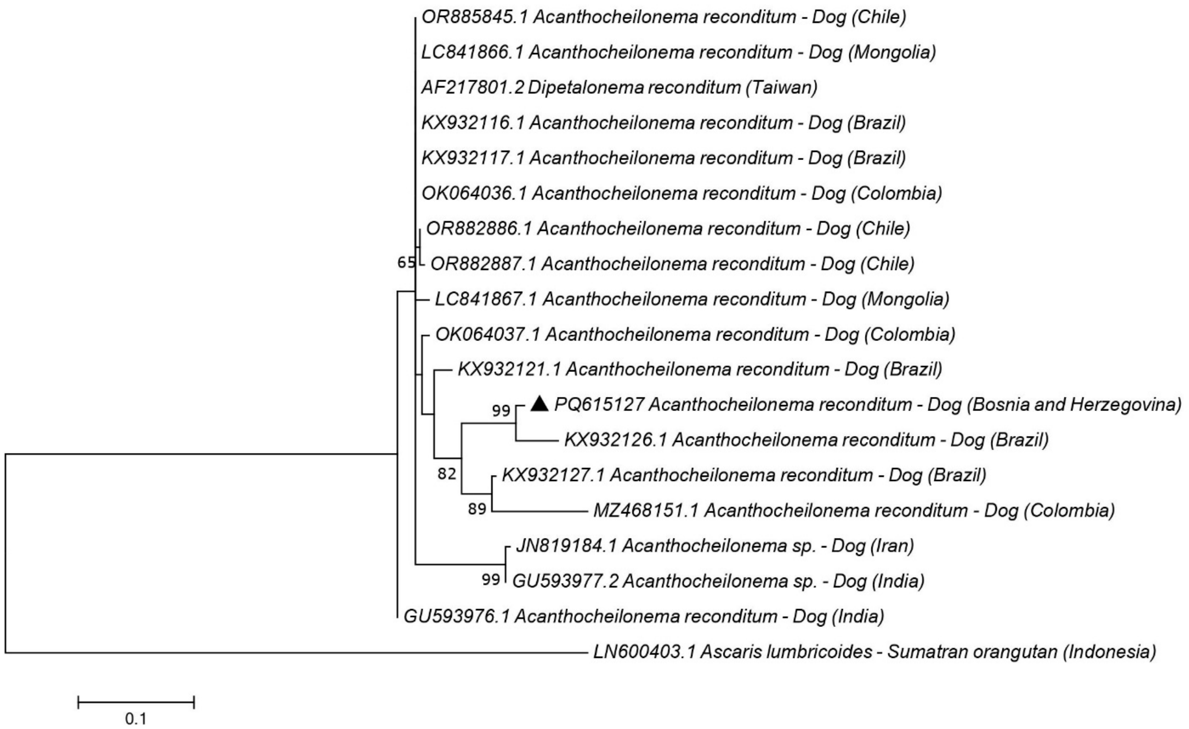

Figure S1. Phylogenetic tree of *Acanthocheilonema reconditum* isolates (indicated by ▲) and related species.
